# Biomolecule-Enabled Liquid Separation Membranes: Potential and Recent Progress

**DOI:** 10.3390/membranes12020148

**Published:** 2022-01-25

**Authors:** Faiz Izzuddin Azmi, Pei Sean Goh, Ahmad Fauzi Ismail, Nidal Hilal, Tuck Whye Wong, Mailin Misson

**Affiliations:** 1 Advanced Membrane Technology Research Centre, School of Chemical and Energy Engineering, Faculty of Engineering, Universiti Teknologi Malaysia, Johor Bahru 81310, Malaysia; muhammadfaizizzuddin.graduate@utm.my (F.I.A.); afauzi@utm.my (A.F.I.); wongtuckwhye@utm.my (T.W.W.); 2 NYUAD Water Research Center, New York University Abu Dhabi, 129188 Abu Dhabi, United Arab Emirates; 3 Biotechnology Research Institute, Universiti Malaysia Sabah, Kota Kinabalu 88400, Malaysia; mailin@ums.edu.my

**Keywords:** membrane modification, liquid separation, biomolecules, surface coating, surface grafting, biomimetic

## Abstract

The implementation of membrane surface modification to enhance the performance of membrane-based separation has become a favored strategy due to its promise to address the trade-off between water permeability and salt rejection as well as to improve the durability of the membranes. Tremendous work has been committed to modifying polymeric membranes through physical approaches such as surface coating and ontology doping, as well as chemical approaches such as surface grafting to introduce various functional groups to the membrane. In the context of liquid separation membranes applied for desalination and water and wastewater treatment, biomolecules have gained increasing attention as membrane-modifying agents due to their intriguing structural properties and chemical functionalities. Biomolecules, especially carbohydrates and proteins, exhibit attractive features, including high surface hydrophilicity and zwitterionic and antimicrobial properties that are desired for liquid separation membranes. In this review, we provide an overview of the recent developments in biomolecule-enabled liquid separation membranes. The roles and potentials of some commonly explored biomolecules in heightening the performance of polymeric membranes are discussed. With the advancements in material synthesis and the need to answer the call for more sustainable materials, biomolecules could serve as attractive alternatives for the development of high-performance composite membranes.

## 1. Introduction

The water contamination crisis is one of the main issues in arid regions in the world such as the Middle East and North Africa [1]. Water pollution caused by agricultural residues, sewage, and industrial waste has exacerbated this problem [2]. With the rapid increase in demand for fresh and clean water resources, the shortage of fresh water has become a key issue affecting the economic and social development of many countries. The alarming conditions have urged the implementation of various water reclamation processes to produce fresh water to fulfil the current needs. Besides traditional urban water treatment, desalination has also become a promising alternative for the reliable supply of fresh water [3]. Among the many wastewater treatment and desalination technologies, membrane technology has been used as a main treatment technology in the current water treatment and desalination industries. There are several types of membrane-based separation, but the popular separations are pressure-driven and osmotically driven membrane processes [4]. Both processes have been used in a broad range of applications, particularly in the chemical industry and the wastewater treatment industry. The process requires a semi-permeable membrane that will act as a barrier to prevent larger particles or molecules from feed solutions from penetrating the membrane pores to produce permeate water [5].

The typical types of pressure-driven membranes are reverse osmosis (RO), nanofiltration (NF), ultrafiltration (UF), and microfiltration (MF) [6]. In general, MF and UF are mainly used to eliminate large particles, colloids, and effectively remove bacteria found in feed solutions [7]. These separations are commonly found in the food industry, pharmaceutical products, pretreatment, and effluent treatment before NF or RO processes [8]. NF is equipped with a small pore size <2 nm and low molecular weight cut-off (MWCO) at 100–2000 Da and typically charged surfaces in aqueous condition [9]. NF can eliminate bi- and tri-valent ions from water such Mg^2^^+^, Ca^2^^+^, and Al^3+^ at an efficiency rate of about 80–85% and monovalent ions such as Na^+^, K^+^, and Cl^−^ at an efficiency rate of 40% [10]. RO membrane technology has become a popular choice in desalination, drinking water production, and wastewater treatment due to its efficiency in removing almost all classes of pollutants to produce high-quality permeate water [11]. The operating pressure applied for NF and RO is much higher compared to MF and UF, but the overall permeability is much lower than that of MF and UF membranes.

The osmotically driven membrane process, typically represented by forward osmosis (FO), is another type of membrane-based separation that is based on osmotic driving forces. Compared to its pressure-driven counterpart, FO is less energy intensive and studies show that membrane fouling on FO is also relatively less severe [12]. With high osmotic pressure across the membrane, FO is able to achieve high water flux and water recovery [13]. FO has been exploited in the food and pharmaceutical industries due to its capability of retaining the physical properties of the feed without having any significant changes since the feed is not pressurized [14]. 

Despite the steady growth of membrane-based processes in the separation market, constant efforts have been made to improve their performances and address their bottlenecks. The properties of membranes, known as the heart of the entire membrane-based process, have been improved through many innovative ways to divest the inherent issues and limitations [15]. Among the strategies used for the optimization of membrane properties, membrane modification is one of the most explored strategies, owing to its versatility and effectiveness in rendering the desired functionalities [16]. Currently, membrane surface modification has been pursued to alter and fine-tune the physio-chemical properties of the membrane. A membrane with carefully tailored properties ensures the efficient performance of the separation process. Tremendous works have been undertaken to achieve this purpose, such as by incorporating nanomaterials onto a polyamide (PA) layer of thin-film composite (TFC) membranes to increase the membrane separation performance and improve the antifouling property [17], by using the co-solvent-assisted interfacial polymerization (CAIP) method to eliminate the trade-offs between salt rejection and water permeability [18], and by introducing some additives such as polyethylene glycol (PEG) and polyvinylpyrrolidone (PVP) during the casting process to enhance the pore formability and surface hydrophilicity of the membrane [19]. 

The main idea of surface modification is to improve surface characteristics such as morphology, surface composition, and surface charge without causing significant effects on the bulk properties of the membrane. Surface modification can be divided into two major categories, which are physical modification and chemical modification [20,21,22]. For physical modification, the membrane surface and modifier materials are bound together by non-covalent interactions such as hydrogen bonding, van der Waals forces, and electrostatic interactions [23]. The most common techniques applied are surface coating and layer-by-layer assembly (LbL). Surface coating is a technique to take over the surface composition in which the chemical additives or agents are attached to the membrane surfaces. This process changes the surface performances’ focus on the active layer. It is considered a simple, feasible, and eco-friendly method for surface modification. 

For the chemical modification approach, the modifying chemical is attached together with the surface active layer via strong covalent bonds, which normally results in better structural and chemical stability [24]. Surface grafting through chemical or radiation approaches is the typical technique used for chemical modifications of the membrane surface [25]. During chemical surface grafting, various chains of hydrophilic moieties and functional groups can be introduced permanently onto the membrane surface [26]. Surface grafting can be classified into two techniques, which are “grafting to” and “grafting from” [23,27]. “Grafting to” involves the direct grafting of preformed functional groups’ chemical additives onto the active layer of the membrane surface via a covalent reaction to improve the membrane surface. The “grafting from” technique allows the monomer to grow from the grafting sites to create new side chains with simple straight-line length onto the surface composition. Grafting-from has been commonly accomplished by the atom transfer radical polymerization (ATRP) technique [23].

In parallel with the development of various membrane surface modification techniques, attention has been dedicated to the exploration of new modifying agents for membrane modification. Currently, membrane surface modification has been mainly performed through the introduction of a wide range of nanomaterials. Nanomaterials with various functionalities have been proven to increase water flux, membrane hydrophilicity, mechanical reliability, salt rejection, and chemical stability of the resultant nanocomposite membrane [28]. Many inorganic-based nanomaterials such as metal and metal oxide nanoparticles have been extensively used as modifying agents of polymeric membranes to form a new class of membranes known as nanocomposite membranes [29]. For example, titanium dioxide (TiO_2_) stands out due to its unique properties, which include low toxicity, photocatalytic properties, and antifouling properties [30]. Mansourpanah et al., proposed TiO_2_ on TFC membranes through the interfacial polymerization process to compare the performances between the presence and absence of UV irradiation towards membrane separation and surface properties of the modified membrane [31]. 

Metal oxides such as Ag, Cu, ZnO, and Mg(OH)_2_ have been classified as nanomaterials incorporated into the PA layer that are broadly used to enhance antifouling in thin-film nanocomposite (TFN) membranes. Yin et al., demonstrated silver nanoparticles (AgNPs) onto TFC membranes via surface grafting, incorporating cysteamine as bridging chemical to create covalent bonding with AgNPs to improve silver-containing modified membranes and enhance anti-biofouling properties [32]. Ben-sasson et al., introduced copper nanoparticles (CuNPs) on the PA TFC RO membrane surface, synthesizing polyethyleneimine as a capping agent via electrostatic interactions to reduce bacterial attachment to the membrane surface and enhance the antifouling properties of modified membranes [33]. Carbon-based nanomaterials such as graphene and its derivatives have attracted much attention in the past decade in this field. Choi et al., attempted graphene oxide (GO) nanosheets on PA TFC RO membranes via the LbL assembly method to enhance the antifouling properties and chlorine resistance of the modified membranes [34]. Tousley et al., demonstrated GO nanosheets on TFC PA RO membranes via chemical coupling with the PA layer to allow efficient antimicrobial properties without having any trade-offs in separation-modified membranes [35].

Another class of modifying material that is also gaining popularity is biomolecules. The applications of biomolecules cover many areas in human life, especially in the therapeutic area, and they are also involved in the water purification process. Biomolecules are also considered eco-friendly, and they have a less negative impact in terms of pollution. Some examples of biomolecules that have been prominently used for membrane modifications are amino acids, sugars, polysaccharides, as well as monosaccharides. Biomolecules such as enzymes has been attempted as the modifying agent of polymeric membranes in some early work, where a change in the surface hydrophobicity/hydrophilicity was observed [36].

**Figure 1:**
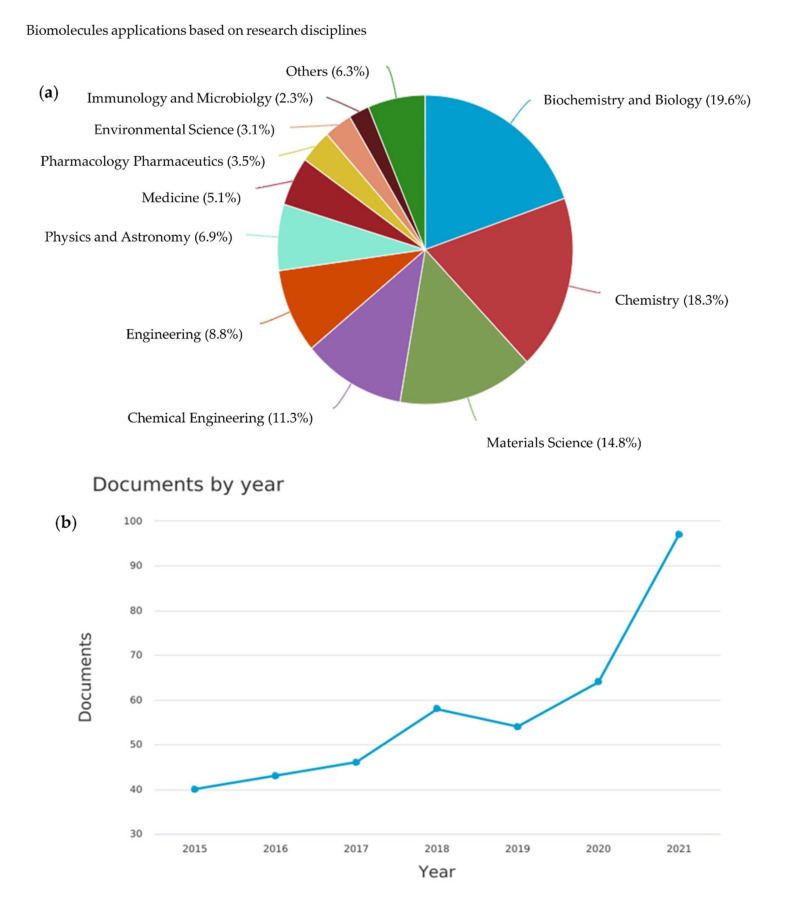
(**a**) The breakdown of the application of biomolecules based on the research disciplines. (**b**) The number of articles published related to the application of biomolecules for water and wastewater treatment.

Biomolecules have been applied in many disciplines, as shown in Figure 1a. Based on Scopus data, biomolecules have been actively investigated in fields of chemistry, especially in biochemistry, chemistry, and material science, accounting for almost 54% of total articles published. An increasing trend in the number of scholarly publications has been observed in the past 5 years, as shown in Figure 1b. It can be deduced that biomolecules have stimulated great interests in many disciplines, including in membrane modification and wastewater treatment.

The membranes modified with biomolecules exhibit attractive properties for liquid separation [37,38]. Biomolecules are endowed with some unique features that are desirable for membrane modification [39]. A grafting of zwitterionic polymer amino acids on the membrane active layer enhanced the antifouling property due to the presence of hydrophilic moieties on the membrane surface [40]. Aquaporin protein structures possess a special ability to offer their water permeability in membrane modification as biomimetic membranes [41]. Over the past few years, many studies have been devoted to investigating the effectiveness of biomolecules in enhancing the physio-chemical properties of polymeric membranes used for liquid-based separation. Saiful et al., reviewed the potential of chitosan as the modifying agent of FO membranes to achieve a high rate of salt rejection, metal removal, and bacteria resistance, and to meet the quality standards for drinking water [42]. Recently, Sharma et al., provided an overview of the application of biomimetic nanofiltration membranes with emphasis on the reproducibility, large-scale fabrication, and potential commercial application of the composite membranes [43]. The versatility of mussel-inspired polydopamine (PDA) in modifying polymeric membranes applied for wastewater treatment membranes has been highlighted by Yan et al. [44].

The existing reviews on the modification of liquid-based membranes provide a comprehensive discussion on a broad range of strategies to address issues with certain surface modification approaches, but the use of biomolecules in this regard has not been discussed in depth. A better understanding is vital for the identification of suitable biomolecules as modifying agents and the directions for further optimization. With this review, we aim to provide insights into the potential of exploiting the special features of biomolecules for the preparation of modified membranes with better liquid-separation performance. The commonly used biomolecules are first discussed based on their physio-chemical properties. The modification techniques including coating, layer-by-layer self-assembly, grafting, and atom transfer radical polymerization (ATRP) within the PA RO layer are discussed for every type of biomolecules with their distinctive performances. The challenges and future perspectives in this field are highlighted to provide guidelines and research directions to facilitate the development of modifying agents for RO membranes.

## 2. Biomolecules as Membrane Modifying Agents: Classification and Unique Features

A biomolecule is defined as any substance that is produced by cells and living organisms [45]. There are four major types of biomolecules, which are carbohydrates, fats, proteins, and nucleic acids. The chemical, mechanical, and biological properties of biomolecules constitute crucial features in the development of distinctive biomaterials [46]. One of the potential abilities that biomolecules possess, antifouling, encourages the design and development of surface modification techniques. In this section, we discuss the different classifications of biomolecules and the properties of some of the most commonly used biomolecules for the modification of liquid-based membranes. 

### 2.1. Protein

**Figure 2:**
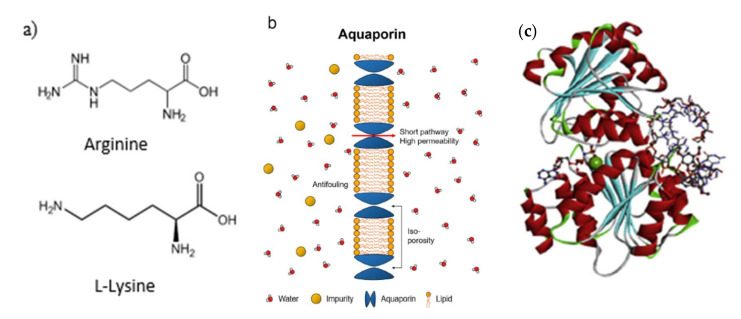
Examples of (**a**) common amino acid; (**b**) aquaporin [49]; (**c**) enzyme [50].

A protein monomer is called an amino acid and it is divided into 20 different groups of side chains. An amino acid consists of two distinct functional groups, which are the carboxyl group and the amino group. Figure 2 shows examples of protein-based molecules. Amino acids, aquaporin, and enzymes are the three major types of protein molecules that have been used for membrane-based separation. Amino acid has proven to be a promising surface modifier for PA thin-film composite (TFC) membranes. Due to the abundance of amine carboxyl functional units, they exhibit elevated surface hydrophilicity in addition to antibacterial actions. Amino acid is known as a zwitterionic polymer, which is a compound that does not have an exact electric charge but possesses different positive and negative charges, known as cationic and anionic, which are able to bind water molecules by electrostatic interactions [47]. It is also able to create a hydration layer that is more physically stable and acts as a membrane barrier that can alleviate the protein adsorption into the membrane [22,48].

L-arginine (Arg) consists of a variety of functional groups in its molecular chains, such as α-amino group, α-carboxylic group, and guanidine group, which are classified as hydrophilic groups and have notable steric hindrance. The hydrophilic groups are flexible and effective without having any influence on the structure of the membrane surface, and they promote extra hydrophilicity on the modified membrane. With the feature of high hydrophilicity and biomaterial zwitterion, the grafting of Arg may enhance the formation of a hydration layer on the membrane surface that can improve the driving force of water molecules to pass through the membranes. Chen et al., incorporated Arg onto the membrane surface to enhance the surface hydrophilicity and antifouling properties. The Arg-modified membrane exhibited strong repulsion towards negatively charged foulants [51]. 

Aquaporin (AQP) is a protein-based molecule known as “the plumbing system for cells”. In biological membranes, AQP proteins provide the most efficient method for allowing water across osmotic pressure gradients [52]. AQPs allow water molecules’ passage at a high rate (up to 10⁹ water molecules s^−^¹) and the molecule virtually rejects any molecule [41]. Its ability to promote high water permeability while retaining favorable salt content is a property that is seldom found in other molecules, and thus, AQPs serve well as biomimetic membranes [53]. AQP is also known as a membrane protein in the cells of organisms, particularly for its high water permeability and rejection of protons, ions, and neutral solutes [54]. Hence, theoretically, an AQP-based membrane could show a more favorable performance compared to conventional NF or RO membranes [55].

As AQPs are part of the integral membrane proteins, AQPs must be integrated into specific amphiphilic carriers such as lipids or block copolymers to maintain their unique functions in an aqueous environment. The amount of AQPs in the membrane would have a significant effect on the performance as more AQPs would contribute to the high water flux. A liposome or polymersome encapsulated with an AQP will condense into a thin plate-like layer upon interaction with specific substrates. Polymersomes or proteoliposomes also refer to these vesicles [56]. AQPs play a crucial role in facilitating the transport of water molecules, as they would be incorporated into a thin layer formed by the fusion of vesicles as a selective layer. The method was demonstrated immobilizing proteoliposomes on membrane surfaces, and increasing the concentration of proteoliposomes able to enhance the membrane performance [57]. By using a layer-by-layer technique, this could be constructed by polyelectrolytes.

An enzyme is a protein-based molecule produced by all living organisms. Enzymes are well known as biocatalysts that participate in a wide range of metabolic processes. The size of enzymes typically ranges from 100 to more than 2500 amino acid residues [58]. They can be obtained from different sources, such as animals and plants. For animal-based enzymes, the most common enzymes are pancreatic lipase, rennin, trypsin, lactase, and amylase. Animal enzymes mainly focus on digesting protein with minimal presence of starch and fat-digesting capabilities [59,60,61]. For plant-based enzymes, there are four enzymes that can be identified, which are protease, amylase, lipase, and cellulose. They are known as enzyme-rich and easy to consume, and ultimately conserve the full ability of the enzymes [62,63,64].

**Figure 3:**
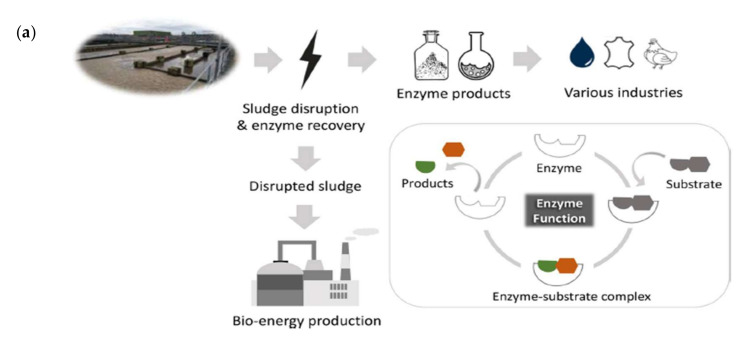

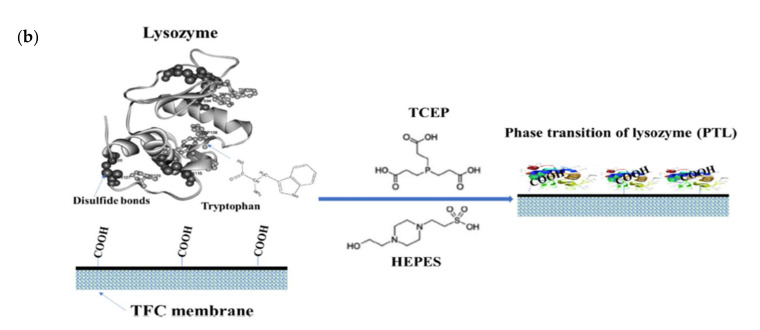
(**a**) The roles of enzymes in industrial applications [65]. (**b**) Illustration of phase transition of lysozyme on PA membrane surface [70].

Enzymes are able to promote advantages for inorganic catalysts, effectively reacting under mild conditions [65]. They have been applied in a broad range of industrial fields, such as in wastewater treatment plants and food processing. They are also known as antimicrobial agents and the most typical antimicrobial enzyme is lysozyme [66]. Lysozyme is produced by humans and animals, and constitutes part of the immune system. Zhou et al., implemented lysozymes and papain in sewage sludge anaerobic digestion in the pharmaceutical field to remove triclosan, an antibacterial agent susceptible to enzymatic degradation [67]. The results showed that lysozymes and papain gave the best performance with enhanced contaminant removal by improving accessibility and biodegradation. Barber et al., recently demonstrated oxidoreductase enzymes cross-linked with polyethylene glycol (PEG) to achieve organic micropollutant degradation in wastewater treatment [68]. Roesink et al., reported the presence of enzymes able to enhance fighting micro-pollutants in water treatment, either by breaking them down or adjusting the pollutant properties to help clean the water [69]. Figure 3a shows an illustration of enzymes that have been used in industrial applications. The waste product of biological wastewater treatment known as activated sludge (AS) comprises microbial biomass that decomposes organic materials. As a result, enzyme recovery from AS is a feasible alternative to industrial enzyme production. Tian et al., introduced lysozymes on a PA membrane surface via simple surface coating and improved the antibacterial properties to mitigate bacterial attachment on the membrane surface [70]. As shown in Figure 3b, lysozyme consists of four pairs of disulfide bonds and six tryptophan residues. One of the amino acid groups reacts with tris(2-carboxyethyl) phosphine (TCEP) and 2-[4-(2-hydroxyethyl)piperasin-1-y] ethanesulfonic acid (HEPES) buffer to form phase-transition lysozyme (PTL)-modified membranes, which exhibit immense bacterial reduction and are able to hinder trade-offs between water flux and salt rejection.

Schulze et al., incorporated the capability of pancreatin via immobilization on PES membranes to evaluate the membrane surface cleaning and membrane separation performances [71]. Pancreatin is a type of enzyme consisting of lipase, protease, and amylase and is broadly utilized in the food and pharmaceutical industries. They reported that the immobilization of pancreatin with different classes of enzymes could mitigate different types of fouling problems. Wong J. et al., reported that enzymes such as lignin peroxidase, soybean peroxidase, and horseradish peroxidase hold great potential in their applications for dye wastewater treatment, where they can offer positive outcomes in eliminating toxic materials [72].

### 2.2. Carbohydrate

**Figure 4:**
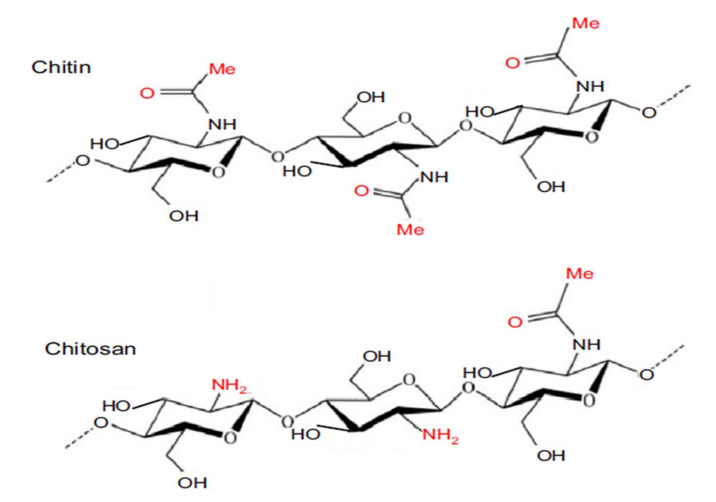
Chemical structure of chitin and chitosan [74].

A carbohydrate is mainly composed of molecules containing carbon, hydrogen, and oxygen with the general formula C_6_ H_12_ O_6_. Carbohydrates are one of the most abundant organic substances in nature. They can supply energy, are essential constituents to all living things, and are classified as biomolecules. They can be divided into several types of molecules, but those that have been widely used for liquid-based membrane modification are mainly chitosan and cellulose. Chitosan is known as a natural biomaterial that typically can be found in biomedical applications. The process of forming chitosan occurs when chitin undergoes the deacetylation process by hydrolysis of the acetamide groups with the presence of concentrations of NaOH or KOH at a high temperature, 100 °C. The reaction commonly requires heterogeneous conditions [73]. Figure 4 shows the chemical structure of chitin and chitosan. Typically, amino groups are far more active compared to the hydroxyl group with the following order: C_2_-NH_2_ > C_6_-OH > C_3_-OH [74]. After cellulose, it is also known as the second most efficient natural polymer. Chitosan is made of glucosamine and a fraction of acetylglucosamine residues [75]. Chitosan has a wide array of applications utilized to solve different issues with respect to nature, accentuated more in biomedical engineering [76]. Chitosan possesses favorable solubility, biocompatibility, and degradability [77]. Chitosan can also act as a multifunctional polymer with different purposes due to its unique traits such as hydrophilicity, harmlessness, low cost, biodegradability, straightforward chemical derivatization, and being equipped with effective adsorbents of metal ions, known as chelating agents. Besides its inherent biosorption, chitosan’s adsorption capability and selectivity could be improved via chemical and physical modifications of the amine and hydroxyl groups [78].

Nowadays, studies are focused on the development of chitin or chitosan in environmental fields, which includes the removal of dyes, chemical waste, and metal ions from wastewater [79]. Chitosan is one of the chelating agents that can adsorb a metal ion abundantly from an effluent system and this has intrigued researchers to further study the potential of chitosan, especially with its feasibility and versatility. It has been demonstrated that chitosan can efficiently alleviate particulate and dissolved contaminants by coagulation–flocculation processes, leading to several techniques such as charge neutralization, bridging, and electrostatic patch [80]. 

Chitosan is also able to remove oils, grease, and particulate matter from wastewater streams. In water treatment, membrane modification with the presence of chitosan has shown promising results [81]. Due to the availability of free amine functional groups, it reacts with negatively charged surfaces and enhances the antifouling properties. It is a hydrophilic polymer used to adjust hydrophobic membranes such as poly(vinylidene fluoride) (PVDF) and polystyrene to enhance their hydrophilicity properties [82]. The presence of chitosan in membrane surface modification may improve the uniformity and stability of the coating layer; for example, the surface morphology, surface charge, and wettability of the modified membranes were demonstrated in various studies. 

Another type of biomolecule-based carbohydrate is cellulose. It has been classified as the most abundant biomaterial on the planet and is a nearly unlimited source of raw material in the production of eco-friendly and sustainable bioproducts [83]. It can only be found in bacteria and plant cells and is present in the cell walls. It plays a vital role in providing strength and structure to plants. With the latest technology, the production of nanocellulose and its various uses are gaining researchers’ attention. The advantageous properties include the addition of water permeability, increased surface hydrophilicity, and high resistance to biofouling [84,85,86]. Nanocellulose-based materials that are mechanically stable with antifouling properties are also developed for water filtration. Nanocellulose can be manufactured on a large scale with feasible costs using various methods and is also a natural biopolymer capable of being prolonged. The main properties of nanocellulose are high surface area, renewable, biodegradable, high chemical resistance, surface functionalization, and water stability [87]. The mechanical properties of nanocellulose are affected by the high surface area, where the abundancy of hydroxyl group and hydrogen bonding is also greatly increased.

Nanocellulose can be further divided into three main different types, which are cellulose nanocrystals (CNCs), cellulose nanofibrils (CNFs), and bacterial nanocellulose (BC). CNC, known as cellulose nanowhisker, is a nanomaterial of cellulose derived from natural sources such as plant fiber [83,87,88,89]. CNCs have desirable properties including biodegradability, stability, and high strength with a large surface area for functionalization. In terms of surface modification properties, CNCs are known as a hydrophilic, chemically stable, highly crystalline, and non-leachable nanomaterial with a large surface area, making them promising modifying agents in membrane fouling reduction. They can be chemically altered to enhance their solubility and chemical stability, giving them new abilities such as magnetism, adsorption, separation, and catalysis. This cellulose composite has the capability to remove heavy metallic ions, oils, and even pesticides from aqueous systems via adsorption.

CNFs have a high surface area and many hydroxyl groups available for surface grafting compared to the cellulose nanocrystals. CNFs also provide increases in membrane tensile strength by the addition of small amounts of CNF [85]. These nanocelluloses are abundant and also provide novel applications in a broad range of fields, which can be attributed to their chemical and physical functionalities. BC is equipped with a high capacity for binding water molecules, biodegradability, and sustainability to produce an ultrafine membrane that makes it more stable in water [90,91].

### 2.3. Polydopamine

**Figure 5:**
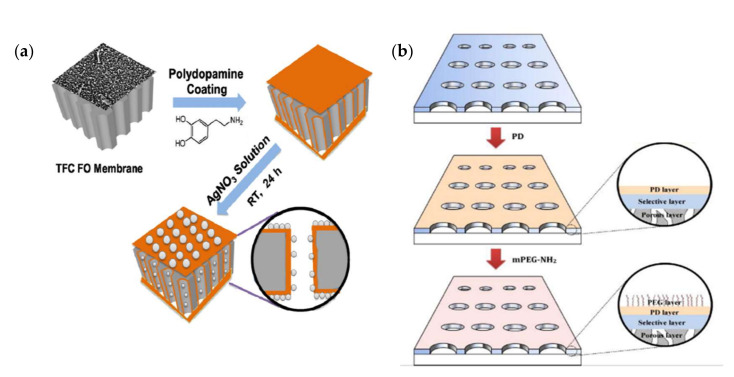
Schematic illustration of (**a**) self-polymerization of dopamine on TFC FO membrane followed by in situ deposition of silver nanoparticles [99]; (**b**) surface coating of PDA layer followed by grafting of hydrophilic polyethylene glycol group [103].

Polydopamine (PDA) is known as a “bio-inspired” material and it has been widely used for the surface modification of many types of liquid-based separation membranes [92]. PDA can attach to almost all kinds of substrates and thus has been used for surface coating due to its material-independent coating ability [93]. PDA has been widely used for the coating of various materials, including metal oxides with low surface energy [94]. The straightforward coating process and the unique features give PDA numerous practical uses. PDA contains an abundant functional group of amines and high catechols that contributes to its strong interfacial adhesion [95]. Therefore, this feature has been further exploited to immobilize different functional species such as biomolecules and long-chain molecules onto different types of surface [96]. A number of studies have described the broad use of this biocompatible material, in terms of energy, water purification, and even in the biomedical industry [97]. When introduced to the membrane surface, the PDA coating formed through the self-polymerization of dopamine has a negligible effect on the integrity of the membrane structure and can feasibly provide a large number of active surface groups on the membrane surface [98]. The surface reactive groups can be used as active sites for the subsequent grafting of other functional molecules. Figure 5a shows the schematic illustration of the surface coating of PDA, which served as the deposition site for the in situ formation of silver nanoparticles on the top and bottom surfaces of TFC membranes in one study [99]. 

PDA demonstrates promising hydrophilicity associated with the presence of functional groups of amines and hydroxyls within it [100,101]. Due to the nature of PDA, it can be flexibly used to improve the hydrophilicity of many types of hydrophobic membranes which normally suffer from the fouling issue [102]. As shown in Figure 5b, Mccloskey et al., first polymerized dopamine on the surface of a polymeric membrane, followed by the incorporation of PEG via surface grafting to improve surface hydrophilicity and fouling resistance in oil-in-water emulsion conditions [103]. Through this surface modification, the PDA deposition was found to enhance fouling resistance, while grafted PEG was able to retain all polymeric membrane separation performances. Muchtar et al., reported that PDA has high resistance to UV due to the rich content of catecholamines (hydroxyl and amine groups), which are capable of preventing UV free radicals from penetrating the membrane surface [104]. Shah et al., observed that PDA coated on hydrophobic PVDF UF membranes could enhance the water permeability and antifouling properties [105].

## 3. Modification Strategies

Membrane morphology, membrane composition, and chemistry of active layers are related to the hydrophilization, water permeability, surface roughness, and foulant resistance of the membrane [106]. As mentioned earlier, surface modification of membranes can be conducted via physical or chemical surface modification [107]. This section provides an overview of the physical and chemical modification approaches to introduce biomolecules to the membrane surface or within the membrane matrix.

### 3.1. Physical Modification

Surface coating is a relatively simple and straightforward procedure for physical surface modification in which the layer deposition takes place on the membrane surface. A wide range of functional materials with desired properties, such as wettability, surface roughness, dispersibility, and biological activities, can be introduced to the membrane surface via surface coating. Typically, the surface coating process involves the immersion of a PA film in the coating solution and then evaporating the residual solvent at a medium temperature to strengthen the coating solution and create a thin coating layer on the active layer of the membrane [108,109,110]. In deciding the physiochemical characteristics of the modified membranes, the coating content and conditions are critical. Shahkaramipour et al., reported that the surface charge and roughness were reduced without a negative impact on salt rejection and water flux efficiency when PDA was introduced via surface coating [111]. 

**Figure 6:**
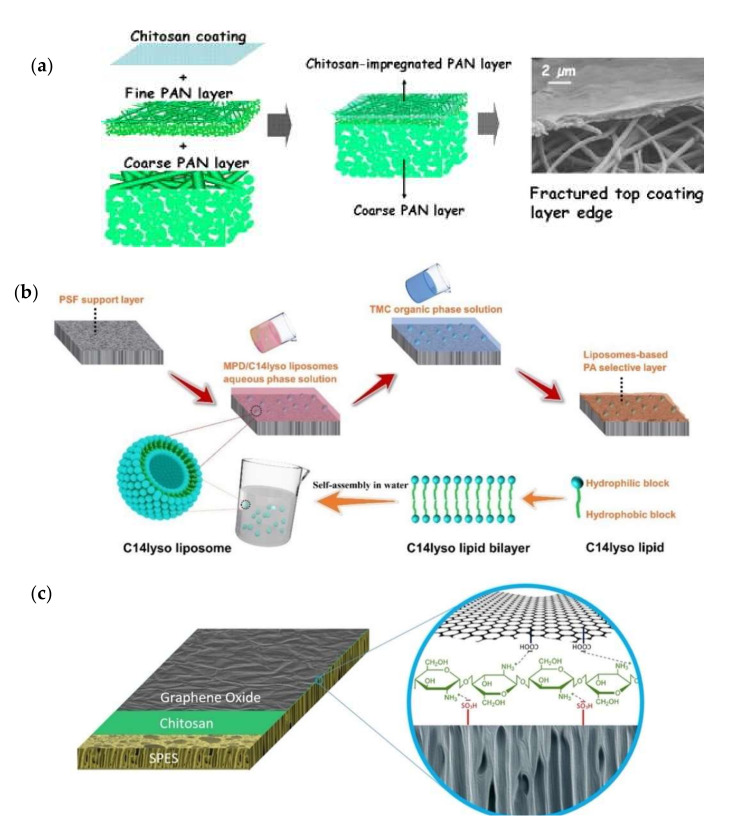
Schematic illustration of the common approaches used for physical modification of polymeric membranes. (**a**) Surface coating of chitosan on electrospun polyacrylonitrile substrate [115]; (**b**) the incorporation of liposomes into the polyamide selective layer of TFC membranes [116]; (**c**) LbL assembly of chitosan and graphene oxide onto sulfonated polyether sulfone substrate [117].

Figure 6a shows an example of surface coating in membrane modification. The materials used for surface coating are typically hydrophilic polymers comprising multiple functional groups such as hydroxyl, carboxyl, and amino groups, or enzymes [112]. Azari and Zou demonstrated the surface coating of L-dopa, which is known as a zwitterionic amino acid found in marine mussels [113], onto RO membranes to alleviate fouling caused by bovine serum albumin (BSA), dodecyl trimethyl albumin bromide (DTAB), surfactants, and alginic acid [114]. Surface coating can be implemented through several techniques such as spin coating and vacuum filtration.

Another extensively used physical surface modification technique is direct blending. Various types of modifying agents, especially inorganic-based nanomaterials, have been introduced into the polymer matrix through direct incorporation during the preparation of polymer dope or during the interfacial polymerization of the polyamide layer. For the former, the preformed modifying agents are added into the dope solution during polymer dope preparation. The state of dispersion of the modifying agent in the polymer dope dictates their distribution and dispersion throughout the as-fabricated membrane. The latter case is a commonly used technique to produce thin-film nanocomposite membranes. The modifying agents can be introduced in the aqueous phase or organic phase of the monomers used for the interfacial polymerization of the polyamide layer. Despite the feasibility of this approach, the conditions must be carefully tailored so that the overall integrity of the polyamide layer is not disrupted by the presence of foreign materials during the polymerization process. Alternatively, the modifying agents can also be incorporated into the substrate of TFC membranes prior to the formation of the polyamide layer.

As shown in Figure 6b, Chen et al., incorporated liposomes into the aqueous phase of the monomer of the polyamide selective layer during the interfacial polymerization to fabricate liposomes-based RO membranes [116]. They observed that the change in the diffusion rate of the m-phenylenediamine monomer from the aqueous phase to the organic phase was slowed down by the existence of the liposomes, especially at high monomer concentrations. The slower diffusion rate is associated with the formation of a thinner polyamide layer. Besides offering support, the substrate of TFC membranes also has favorable effects on the physicochemical and structural properties of the developed polyamide layer. Mamah et al., incorporated a hybrid of chitin nanofibers and palygorskite into the polysulfone substrate of TFC RO membranes [118]. They observed that the hydrophilic modifying agent optimized the substrate structure in terms of its porosity and tortuosity. The incorporation of the hybrid nanomaterial facilitated water transportation by providing more water pathways through the creation of extra void space for water passage.

LbL has been commonly used because of its simplicity in regulating film nanostructure and composition [119]. By tailoring the deposition conditions, LbL offers durable and defect-free deposited layers onto the membrane surface. Typically, the LbL process involves dipping an initially charged substrate such as a positive net surface charge into a dilute aqueous solution of the complementary polyelectrolyte and allowing it to adsorb and “overcharge” the substrate surface [120]. Each layer of the LbL film has a certain thickness at a molecular scale that can be attained in a simple way. The LbL self-assembly is the easiest method to organize nano-layered films. There is no specific equipment or certain polyelectrolytes needed for this technique to be applied. By LbL, film thickness is accurately organized on a nanometer scale and film properties can be improved by changing the polyelectrolyte type. Polyelectrolytes are water-soluble polymers and can favorably increase membrane surface hydrophilicity. With an increasing number of deposited films, the surface charge density will rise; hence, the polyelectrolytes create loops and tails on the substrate surface which can further enhance the hydrophilicity. Figure 6c illustrates the schematic diagram of a membrane substrate assembled with a bilayer of graphene oxide and polydopamine.

Saqib et al., introduced multilayer polyelectrolyte on RO membranes via LbL assembly [119]. The multilayer polyelectrolyte enhanced the antifouling properties of the RO membranes while maintaining salt rejection in the acceptable range. The antifouling ability was improved with the increasing number of layers due to the improved surface hydrophilicity and the reduced surface roughness. Wang et al., assembled multilayer graphene oxide (GO) nanosheets via the LbL approach onto a UF membrane to enhance the surface charge of the membrane. The surface-modified membrane could remove the negatively charged foulants such as polysaccharides and natural organic matters (NOM) [121]. Kwon et al., proposed a new method to overcome the drawbacks of unsatisfactory salt rejection caused by loose structures of polyelectrolytes by developing a cross-linking of MPD and TMC at the molecular level, described as molecular LbL (mLbL), which strengthened the construction of the cross-linked PA layer [122]. The mLbL technique is a more promising approach for RO membrane modification compared to the typical polyelectrolyte LbL multilayers. The mLbL method creates a dense, less tortuous, well-defined PA structure and records high salt rejection compared to polyelectrolyte LbL multilayers. In summary, surface coating and layer-by-layer self-assembly techniques are the most favored techniques for physical modification of the surface membrane by incorporating different types of modifiers mainly on biomolecules such as chitosan and PDA to enhance the membrane separation performance, especially in terms of fouling resistance and flux enhancement.

### 3.2. Chemical Modification

**Figure 7:**
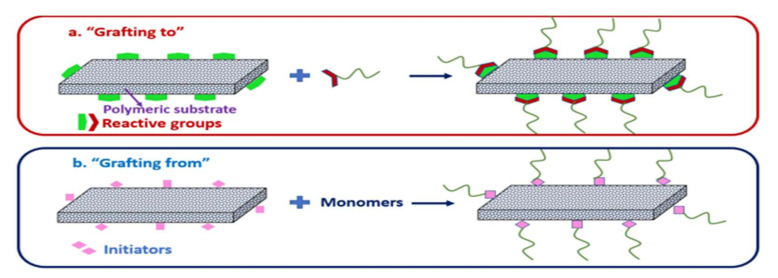
Strategies of surface grafting: (**a**) grafting to; (**b**) grafting from [127].

Surface grafting techniques refer to the attachment of macromolecules or the addition of functional groups of modifiers onto the membrane surface through the establishment of chemical bonding [123]. Grafting has been commonly used to introduce biomaterials onto the membrane surface [23], where it is proven to enhance membrane performance, especially the antifouling properties [124]. Specifically, many studies have applied grafting techniques to modify the membrane surface with amino acids [125]. Grafting commonly uses two strategies, which are known as “grafting to” and “grafting from” [126]. The “grafting to” method is a straightforward technique that can be accomplished in a single step. The polymers carrying reactive functional groups at their end chains are covalently linked to the membrane surface, as illustrated in Figure 7a [127]. In the “grafting from” method, monomers from the surface are used to initiate polymerization with the active functional groups that exist on the membrane surface to form side chains of certain lengths, as illustrated in Figure 7b. For example, Xu et al., demonstrated a grafting to technique with natural amino acids onto PES UF membranes through an epoxy ring opening reaction [128]. Eventually, the modified membranes exhibited enhanced hydrophilicity and antifouling properties due to the presence of the zwitterionic monomers.

Grafting from polymerization takes place upon the creation of active sites on the membrane surface. The generation of reactive sites can be accomplished through different approaches, including chemical redox systems such as potassium persulfate-sodium metabisulfite and oxidants, plasma, and irradiation. Surface grafting through ATRP offers an effective control over molecular weight, preparation of polymers with narrow molecular weight distributions, fabrication of various modifiers, as well as synthesis of hybrid compositions [129]. ATRP can be mediated by redox-active transition metal complexes [130]. The rate of an ATRP is influenced by the rate constant of propagation and the concentrations of monomers and growing radicals. It has been successfully utilized to prepare various copolymers with any desired complex macromolecules structures. ATRP has been used to precisely prepare segmented, block, and graft copolymers. This technique was employed by Mushtaq et al., who introduced different concentrations of 3-sulfopropyl methacrylate potassium (SPMK) onto TFC PA RO membranes via ATRP to render high permeability and antifouling properties [131]. Liu et al., formed a zwitterionic polymer brush layer via the ATRP technique onto TFC PA RO membranes to enhance fouling resistance [132]. The modified membranes exhibited low surface roughness, enhanced surface hydrophilicity, and reduced surface charge due to the presence of both cationic and anionic groups in the zwitterionic polymers. 

Besides ATRP, surface grafting of membranes can also be performed through plasma-assisted or radiation-assisted approaches. Plasma-assisted modification generates both hydrophilic and hydrophobic surfaces by involving various functional groups such as carboxylic, amino, methyl, and hydroxyl groups [133]. Plasma-assisted graft polymerization entails the simultaneous grafting and polymerization of functionalized monomers on the surface material [134]. Under the controlled operating conditions, reactive sites produced from the plasma treatment can promote a free radical polymerization reaction. A functional molecule can be grafted onto a plasma-activated surface from its monomer to form a grafting from polymer brush. Compared to chemical-initiated grafting techniques, plasma-assisted grafting is more cost effective and it reduces the usage of chemicals, as a chemical initiator is not required. Irradiation-induced graft polymerization allows direct crosslinking, viable processing, direct initiation without additives, and a homogeneous reaction system [135]. UV light can be utilized for the surface modification of TFC membranes [136]. The main advantage of UV irradiation-assisted polymerization is the ability to regulate the polymer bond cleavage by optimizing the emission intensity and wavelength of the irradiation [137]. 

## 4. The Roles of Biomolecules in Enhancing Membrane Separation Performances

In this section, we discuss the roles of different classes of biomolecules in advancing the properties of polymeric membranes, either in the form of asymmetrically skinned or thin-film composite membranes, used for liquid-based separations, and elaborate based on some exemplary studies.

### 4.1. Protein-Based Biomolecules

Xu et al., introduced Arg amino acid and PVA onto PA RO membranes via surface grafting to enhance water permeability, chlorine resistance, and antifouling properties [138]. In this surface modification configuration, Arg acted as an antifouling modifier to hinder protein adsorption while PVA possessed hydrodynamic resistance and enhanced the chlorine resistance of the membranes. Upon the surface grafting, the water flux and salt rejection increased from 52.8 L/m^2^h to 57.2 L/m^2^h and from 95.58% to 99.50%, respectively. The presence of grafting PVA-Arg reduced the hydrodynamic resistance of water molecules and enhanced the water flux by facilitating the interaction between water molecules and the membrane surface. In the chlorine stability test, the neat membrane exhibited a rapid decline in salt rejection from 95.58% to 70.94% after soaking in 30,000 ppm NaOCl solution for one hour of while the surface-modified membranes showed a less severe decline in salt rejection from 99.50% to 94.50% under the same conditions. During the fouling testing, the lower DRt indicated better antifouling resistance of the membrane. For overall performance, the Arg functionalized PVA grafted onto RO membranes exhibited promising results in terms of water flux, salt rejection, chlorine resistance, as well as the antifouling properties of the modified membrane.

Shan et al., modified the RO membrane surface of by introducing synthetic zwitterionic monomer 3-(4-(2-((4-aminophenyl)amino)ethyl)morpholino-4-ium)propane-1-sulfonate (PPD-MEPS) and natural Arg by a sequential interfacial polymerization (SIP) method to increase the surface hydrophilicity, water permeability, and antibacterial properties [139]. SIP is known as a direct method to propose functional substances onto the PA surface [140]. With the incorporation of 0.5 wt % solution of these molecules, the water contact angle declined from 60° to 28°, indicating that both molecules have a significant effect on the hydrophilicity of the modified membrane. The water permeability also showed an almost 55% improvement from 2.40 L/m^2^h bar^−1^ to 3.81 L/m^2^h bar^−1^, but unfortunately, there was a clear trade-off in salt rejection as the data recorded a slight declined from 99.2% to 98.5% due to the presence of lower crosslink density caused by the SIP step before heat curing. The long-term antifouling performance tested in 300 ppm of BSA showed that modified membranes had higher water permeability, 3.50 L/m^2^h bar^−1^, which was higher than that of the neat membrane with permeability of 2.40 L/m^2^h bar^−1^. The observation has clearly shown that the grafting of PPD-MEPS functionalized with Arg molecules helps to enhance water permeability as well as antifouling properties. The improvement can be ascribed to the superhydrophilic character and low surface roughness of the modified membranes. 

**Figure 8:**
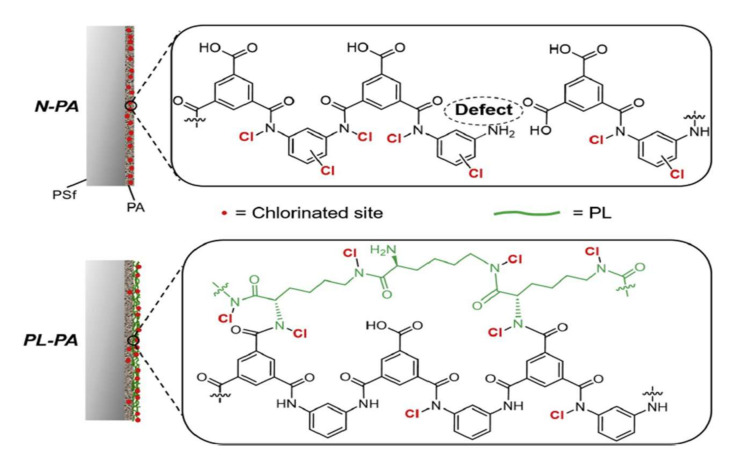
Chemical illustration of chlorination between neat membrane and modified membrane [141].

Zhang et al., introduced the ε-poly-L-lysine (PL) molecule, a homopolymer of L-lysine, via the isopeptide between amino and carboxyl groups to modify TFC RO membranes via the chemical coupling method on the PA layer to improve fouling and chlorine resistance [141]. The contact angle of the modified membranes incorporated with 0.1 to 2.0 wt % was reduced, indicating that the wettability property of membranes improved due to the increase in surface hydrophilicity and reduction in surface roughness. Particularly, the lowest contact angle of 11.6° was recorded for the TFC membrane incorporated with 2.0 wt % of PL. In terms of water flux and salt rejection performance, the neat membrane recorded 43.0 L/m^2^h and 99.5%, respectively, while all the modified membranes recorded a significant increase of 55.0 L/m^2^h in water flux and a slight decreased of 98.9% in salt rejection for the TFC membrane incorporated with 2.0 wt % of PL, implying that the increment was achieved without sacrificing the salt rejection capability. In the antifouling test using negatively charged BSA and positively charged DTAB, the final flux recovery (FRR) significantly increased from 86.6% to 95.2% for BSA fouling and 71.0% to 81.4% for DTAB fouling, when compared with that of the neat membrane. This improvement was ascribed to the improved surface hydrophilicity and the surface smoothness upon the incorporation of 0.5 wt % PL. These surface features minimized the attachment of foulant deposits to the membrane surface. In term of their resistance to chlorine degradation, it was observed that the neat membrane begins to suffer at 1500 ppm of HClO as the “leaf-like” structure starts to diminish, followed by exfoliation of the PA from the PSf substrate. On the other hand, the PL-modified membrane showed no defects at a high concentration of 3000 ppm HClO due to the irreversible N-chlorination that took place on PL chains, as shown in Figure 8. This mechanism protected the PA layer from direct contact with chlorine free molecules. The active chlorine attacked the amidic N-H groups to create N-Cl bonds and eventually, the ring chlorination took place through the intramolecular Orton rearrangement on the PA layer and caused the deterioration of water flux. 

Qi et al., fabricated aquaporin-based biomimetic TFC RO membranes to enhance the durability of surface properties and chemical stability in long-term performance [142]. With the presence of proteoliposomes in aquaporin, the water flux performance improved considerably from 2.68 L/m^2^h for the neat membrane to 4.13 L/m^2^h for the modified membranes. The salt rejection performance was slightly enhanced from 96.5% to 97.2%. During the long-term testing up to 100 days, the normalized water permeability of the aquaporin-modified membrane experienced less severe flux decline compared to the neat membrane, indicating that the antifouling property was improved through the surface modification. The water flux was recovered by 90% after cleaning, suggesting that the fouling was reversible. Interestingly, the aquaporin-modified membranes only needed half the applied pressure compared to commercial membranes to obtain the same flux while producing a sufficient water quality.

**Figure 9:**
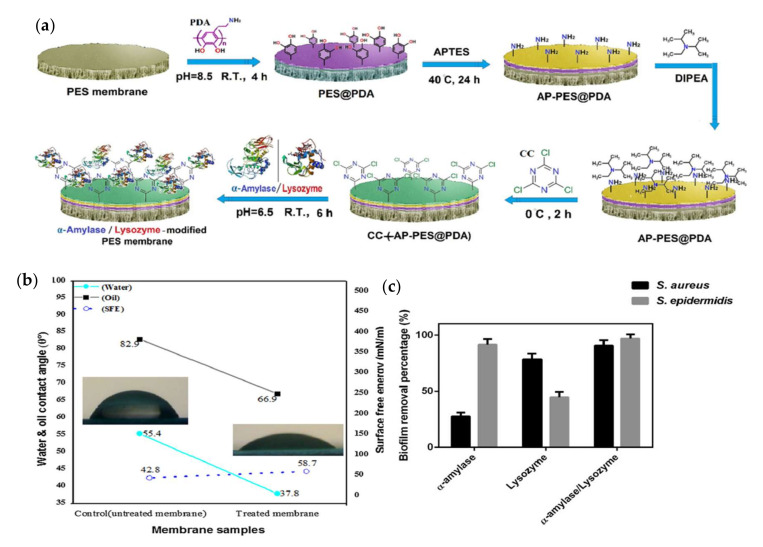
(**a**) Schematic illustration of covalent immobilization of α-amylase/lysozyme onto PES membrane surface. (**b**) Water contact angle between neat and modified membranes. (**c**) The biofilm removal percentage for the membranes modified with amylase, lysozyme, and their hybrids [143].

Mehrabi et al., demonstrated the immobilization of α-amylase and lysozyme enzymes by the covalent binding technique on PDA/PEI-modified PES membranes to mitigate biofilm attachment on the membrane surface, as schematically illustrated in Figure 9a [143]. The formation of biofilms on the membrane surface can be inhibited. α-amylase and lysozyme were selected due to their antimicrobial properties and ability to attack the bacteria [58]. The contact angle was reduced from 55.4° for the neat membrane to 37.8° for the enzyme-immobilized membranes, as illustrated in Figure 9b, which can be attributed to the enhanced surface hydrophilicity. For biofouling performance using *S. aureus* and *S. epidermidis* biofilms, it was observed that the immobilization of α-amylase and lysozyme reduced the bacterial colonies by 87.5% and 94.1%, respectively, as shown in Figure 9c. For the water flux performance, the untreated membrane showed a significant decline from 6722.7 L/m^2^h to 4090.2 L/m^2^h within 60 s due to the build-up of biofilm that caused biofouling on the membrane surface, while the enzyme-immobilized membranes showed a slight decline from 6722.7 L/m^2^h to 6502 L/m^2^h within 60 s due to the action of the enzymes on the biofilm.

Fahrina et al., extracted enzymes from pure ginger and used them as bio-based additives on PVDF membranes prepared through nonsolvent-induced phase separation (NIPS), in which the ginger extract was facilitated by the blending technique into the dope solution to enhance the biofouling resistance [144]. The contact angle of the membrane slightly declined from 92.69° to 84.56° with the increasing ginger concentration in the dope solution to 0.1% *w*/*w*. The water flux was improved with the increment of ginger concentration from 5.07 L/m^2^h to 8.82 L/m^2^h. The membrane hydrophilicity was improved due to the presence of an abundance of hydroxyl groups in the ginger enzyme. The antibiofouling testing conducted using *S. aureus* and *E. coli* indicated that although microbial inhibition action was observed for all the modified membranes, the effect was not significant due to the low concentration of enzymes incorporated into the PVDF membrane.

### 4.2. Carbohydrates

Zhao et al., demonstrated the incorporation of chitosan with the addition of catechol via the surface coating technique on a polyvinylidene fluoride (PVDF) membrane. An effective oil-in-water emulsion separation and enhanced antifouling performance were observed due to the superior hydrophilicity and antibacterial properties of chitosan [145]. Catechol has been used as an alternative to dopamine to form a stable coating. The polymerization reaction took place in the presence of sodium periodate (SP) as an oxidizer between catechol and chitosan. It was observed that the best performance could be obtained using coating solutions of catechol/chitosan at a 1:4 ratio. With the optimum coating, the contact angle of the membrane was reduced from 140° to 8° within 25 s and achieved the highest hydrophilicity among the modified samples. The water flux was tested using peanut oil which indicated that the water flux improved from 254 L/m^2^h for the neat membrane to 428 L/m^2^h for the chitosan-modified membrane, suggesting an improvement of almost 70%. The membrane modified with catechol/chitosan at a 1:4 ratio demonstrated higher removal efficiencies for all types of oil-in-water emulsion systems. It was also observed that the neat PVFD membrane suffered an oil fouling before the modified membrane achieved a superoleophobicity condition with the formation of a hydration layer at the water–membrane interface. Hence, along with the performance shown by the modified membranes in terms of hydrophilicity, catechol/chitosan also enhanced antifouling properties in oil-in-water separation.

Ren et al., immobilized 2-N-propyl sulfonated chitosan (PCS) onto a PVDF membrane that was pre-coated with a PDA layer via Schiff base-assisted deposition to alleviate membrane fouling issues and convey regeneration properties [146]. The water contact angle significantly declined with the coating of PDA, and further plummeted upon the deposition of sulphonated chitosan, indicating that chitosan, which consists of numerous functional groups such as hydroxyl, amino, and sulfonic groups, endowed hydrophilicity to the nanocomposite membrane. The water flux was also immensely improved from 86 L/m^2^h for the nascent membrane to 136.3 L/m^2^h. Similarly, the salt rejection performance of all modified membranes was improved from 48% for the neat membrane to 93% when the chitosan derivative was deposited, with a reaction time of 48 h. The highest BSA adsorption rate of 121.3 μg/cm^2^ was observed for the neat membrane while the lowest rate of 29.3 μg/cm^2^ was exhibited by the modified membrane, suggesting that the PCS incorporation enhanced the antifouling properties of the modified membranes.

Refaat Alawady et al., modified PS membranes with carbon nanotubes functionalized hydroxyl group/chitosan (CNTs-COOH/CS) and multiwalled carbon nanotubes/chitosan (CNTs/CS) via surface coating to improve the rejection of copper, nickel, lead, cadmium, and cobalt [147]. The contact angle was reduced from 73° for the neat membrane to 63.5° and 53.4° for CNTs/CS and CNTs-COOH/CS, respectively. Particularly, the CNTs-COOH/CS membranes achieved high hydrophilicity due to the complementary effect of the carboxylic group present in the CNTs. For water permeability performance, the two chitosan-modified membranes showed improvements, with CNTs-COOH/CS promoting a higher water flux of 3.92 L/m^2^h while CNTs/CS offered a water flux of 1.57 L/m^2^h compared to 0.998 L/m^2^h for the neat membrane. For heavy metal ion rejection, at pH 6–8, both modified membranes showed a high rejection only of copper and lead ions. Meanwhile, at pH 10, both modified membranes achieved the rejection of copper, nickel, lead, cadmium, and cobalt ions aqueous solution. Additionally, CNTs-COOH/CS exhibited almost a 99% rejection rate for all metal ions at pH 10, indicating that the impact of carboxylic group on CNTs significantly enhanced surface hydrophilicity. Overall, this study demonstrated a feasible method for acquiring an abundant performance of heavy metal ion rejection and promoting an eco-friendly environment.

Adeniyi et al., reported the incorporation of CNC derived from sawdust obtained from waste generated by the timber industry [148] during interfacial polymerization of PA TFC RO membranes to enhance surface hydrophilicity and water flux [149]. The results showed that the modified membranes significantly improved in water flux from 7.2 L/m^2^h to 12.7 L/m^2^h, which indicated that the hydrophilicity of the membrane was enhanced due to the presence of the hydroxyl groups contributed by the CNC. Despite having a low trade-off in membrane separation, the modified membranes were able to retain salt rejection at high rate, 97.1%, while for the neat membrane, this rate was 99.7%.

**Figure 10:**
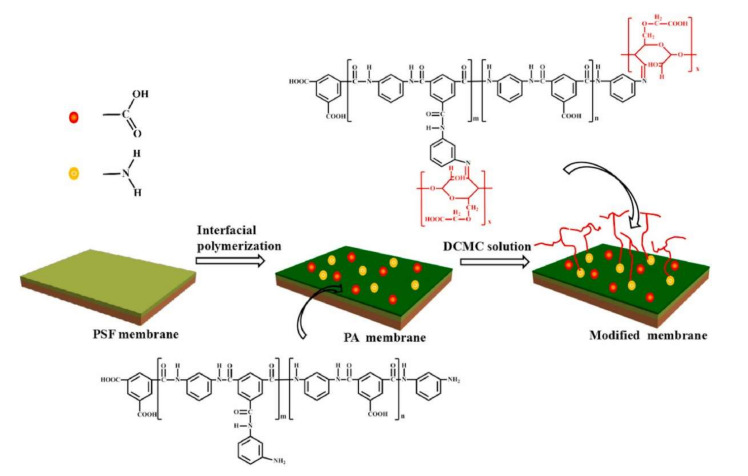
Schematic illustration of the preparation of DACMC grafted on RO membrane [150].

Hu et al., introduced a dialdehyde carboxymethyl cellulose (DACMC) via the surface grafting technique onto RO membranes by a Schiff base reaction, as schematically shown in Figure 10 [150]. DACMC is a derivative of carboxymethyl cellulose (CMC) that possesses an abundance of hydroxyl and carboxyl groups that contribute to hydrophilicity. The antifouling performance was improved due to the alteration in the surface hydrophilicity and surface charge properties of the modified membrane. By increasing the concentration of DACMC, the salt rejection showed a slight improvement of 1.4%, from 97.8% to 99.2%. Nevertheless, the water flux decreased from 35 L/m^2^h to 32 L/m^2^h. The water flux declined due to the enhancement of surface hydrophilicity and the increase in hydraulic resistance from the DACMC grafting process. As the concentration of DACMC increased, the antifouling performance showed an improvement, mainly ascribed to the increased membrane surface hydrophilicity. The surface roughness also plays a vital role in alleviating fouling attachment to the membrane surface. However, further optimization is required to address the trade-off between flux and rejection.

### 4.3. Polydopamine

**Figure 11:**
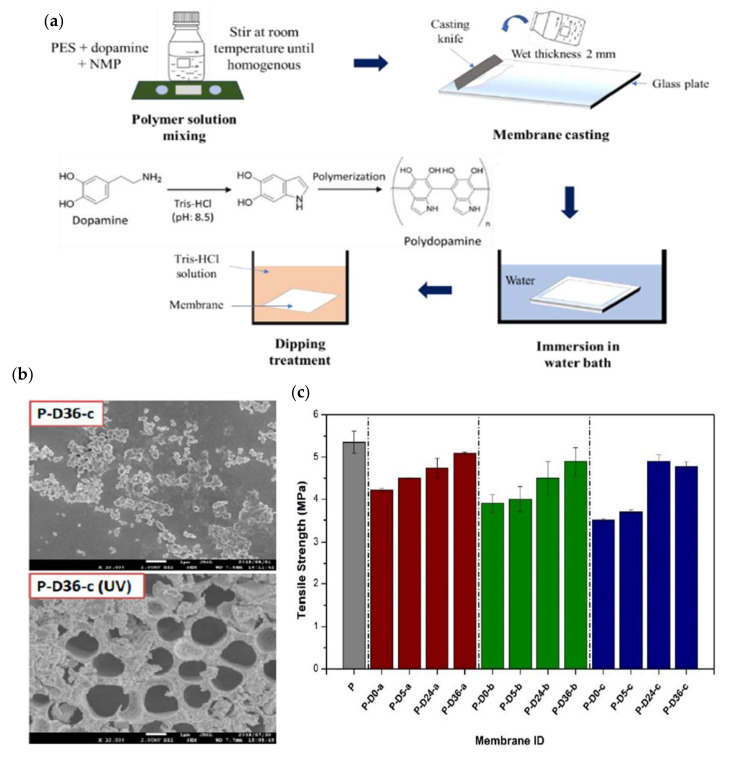
(**a**) Illustration of membrane preparation and dopamine polymerization; (**b**) comparison SEM image between neat membrane and modified membrane; and (**c**) tensile strength performances for neat membrane and all various concentrations of dopamine in modified membranes [151].

Mulyati et al., demonstrated the introduction of PDA onto a PVDF membrane by using a combination of blending and polymerization techniques, as shown in Figure 11a, to improve the antifouling and anti-UV degradation properties [151]. The deposition of PDA reduced the water contact angle from 78° to 47°. The enhanced hydrophilicity of modified membranes was influenced by the functional groups in the PDA, able to alleviate the deposition of foulants onto the membrane surface and improve antifouling properties. In the fouling test using 50 ppm humic acid, the total flux loss was reduced from 50.3% for the neat membrane to 5.3% of the PDA-coated membrane. This improvement was due to the ability of PDA to trigger hydrophilicity and improve the membrane cleaning efficiency. For UV resistance performance, the presence of PDA alleviated the damage on the membrane surface, as evidenced by the SEM image in Figure 11b and proven by the tensile strength in Figure 11c. The tensile strength of the neat membrane reduced from 5.5 MPa to 2 MPa within 7 days due to the porous morphological membrane surface that was vulnerable to free radicals by UV radiation, while the PDA-modified membrane experienced a mild decrease in this tensile strength from 3.7 MPa to 3.2 MPa within the same period, owing to the shielding effect rendered by PDA.

Meirisa et al., modified PES membranes with PDA using a blending technique assisted by hydrogen peroxide (H_2_O_2_) which acts as an oxidizer to create PDA from dopamine through in situ polymerization to enhance the antifouling properties of the modified membranes [152]. The porosity and pure water flux of the modified membranes were enhanced from 2.69% to 11.27% and from 47.06 L/m^2^h to 69.75 L/m^2^h, respectively. The incorporation of PDA increased the water flux by improving the surface porosity of the membrane. The humic acid water flux increased from 27.8 L/m^2^h for the neat membrane to 56.5 L/m^2^h for the modified membrane. However, there is a trade-off between salt rejection and water flux, as the salt rejection of the modified membrane slightly declined from 88.65% to 86.3%. For antifouling performance, the incorporation of PDA enhanced the overall antifouling property of the modified membrane. The flux recovery ratio (FRR) was increased from 66.94% to 94.58%, which indicates the effectivity of the membrane to restore initial water flux after the cleaning process. The total fouling (Rt) of the membrane showed a significant decline from 40.94% to 18.92%, which indicates the ability of the modified membrane to repel the attachment of humic acid on the membrane surface due to the enhanced surface hydrophilicity.

Muchtar et al., blended dopamine into a PVDF dope solution and the membrane was fabricated using the non-solvent induced phase separation (NIPS) method to enhance the antifouling performance [153]. The membrane porosity increased from 12% from the neat membrane to 39% for the membrane incorporated with dopamine. The water contact angle reduced from 77° to 43°, indicating that the membrane surface became more hydrophilic. An immense increase in water flux from 17 L/m^2^h to 111 L/m^2^h was observed. Correspondingly, the humic acid water flux was increased from 9 L/m^2^h to 51 L/m^2^h. Nevertheless, the salt rejection of the membranes was slightly decreased from 95% to 91% due to the enlarged pore size and increased surface porosity. The in situ polymerization of dopamine to form PDA could recover the salt rejection ability due to the formation of a thin PDA layer on the membrane surface. As surface hydrophilicity improved, the modified membrane was able to restore the water flux from 54% up to 92% and 84%.

## 5. Challenges and Future Prospects 

The major challenge of liquid separation processes using polymeric membranes is countering the trade-off between their rejection ability and water permeability. The durability of the polymeric membranes is also of great concern from a commercial point of view. Over the past decade, tremendous efforts have been devoted to investigating the potential of nanocomposite membranes to address the bottlenecks of currently used commercial membranes. Biomolecules are gaining attention in this area due to the special features that are possessed by various types of biomolecules. Biomolecules have been increasingly used as potential modifying agents to enhance membrane separation performance while promoting sustainability. As summarized in Table 1, the potentials of different classes of biomolecules in advancing polymeric membranes used for liquid separation have been investigated in a significant number of works. Compared to the widely reported nanomaterials, including carbon-based nanomaterials such as graphene family nanomaterials, carbon nanotubes, as well as metal and metal oxide nanoparticles, biomolecules are still a relatively new class of materials used for polymeric membrane modification. Therefore, it is necessary to identify their inherent limitations, and substantial efforts are required to address these limitations before biomolecules can serve as alternatives to the well-established nanomaterials for membrane modification.

Despite the variety of biomolecules, not all of them can be suitably used for membrane modifications. Currently, amino acids are one of the most commonly used membrane modifying agents due to their availability, and a relatively simple approach can be used for the introduction of amino acids onto the surface or within the matrix of polymeric membranes. A significant advantage related to amino acids is their small size and zwitterionic properties. Due to the functionality of amino acid, it can be directly grafted onto the surface of a PA layer of TFC with minimum reaction steps and chemical usage. The grafting of a small amount of amino acids imposes negligible effects on transport resistance. This allows the increase in surface hydrophilicity without compromising the water permeability. With the great potential of amino acids for membrane modification, it is worth directing more attention to investigating more types of amino acids. In addition, the potential of dipeptide should also be unleashed. To realize this, it is important to perform a thorough study of the physio-chemical properties of all types of amino acids and their dipeptides, such as their chemical composition, hydrophobicity/hydrophilicity, as well as possible interactions with membrane surface functional groups or membrane polymer chains.

Corresponding to their chemical composition and structure, biomolecules have been mainly used to provide antimicrobial and hydrophilic properties to the resultant nanocomposite membranes. The efficiency of biomolecules in promoting chlorine resistance of the liquid separation membrane has not been extensively explored. The development of a durable membrane that can withstand chlorine degradation is important in many water and wastewater treatment processes, particularly for RO membranes commonly used for desalination. As presented in this review, some biomolecules coated on the membrane surface can act as a sacrificial layer to capture the free chlorine, hence protecting the PA layer from chlorine degradation. Exploring the potential of new biomolecules that can serve multiple purposes, i.e., simultaneously increasing water permeability, antifouling, and chlorine resistance, is an interesting topic that deserves more attention. One feasible strategy to realize this target is to perform pre-functionalization on the biomolecules before they are introduced as membrane modifiers. The desired functional groups such as additional hydroxyl or amine groups can be attached to the biomolecules through chemical or physical interactions. Nevertheless, it should also be pointed out that the pre-functionalization of biomolecules may give rise to some undesirable conditions. With additional functionalization, the biomolecules will become more complicated to execute and the process will also become more time consuming. Furthermore, the additional costs incurred should also be taken into consideration. Alternatively, the biomolecules can also be hybridized with other membrane modifiers, such as carbon-based and two-dimensional nanomaterials. Through this strategy, the biomolecules and the selected nanomaterials can synergistically improve the membrane properties. For instance, the incorporation of antimicrobial nanoparticles such as silver and carbon nanotubes that are coated with hydrophilic biomolecules such as PDA can synergistically improve anti-biofouling properties while achieving high water permeability. The hybridization of two or more modifying agents for polymeric membrane modification is expected to become the future trend in this realm.

## 6. Conclusions

In the past decade, an upward trend has been observed in the application of biomolecules in different research fields. In parallel to the high demands for membrane-based separation in addressing water-related issues, there is an urgent need to develop membranes with high separation performance. In this context, biomolecules emerge as attractive candidates for the development of a new class of bio-based membranes. This review has discussed the approaches and strategies used to introduce biomolecules as modifying agents for membrane modification, due to their various abundant features that help to enhance the membrane surface properties and separation performances. It has been well evidenced that biomolecule-modified membranes demonstrate desired properties due to the hydrophilic and antibacterial properties offered by a wide range of biomolecules. The advantages and bottlenecks of biomolecules have been proposed for future research and improvisation in this field. It is hoped that this review provides researchers in this field with possible ideas concerning the development of membrane modification for wastewater and water purification by providing multiple possible approaches. It is also expected that with the advancements in material science and engineering, the potential of more biomolecules will be unleashed to a greater extent in the near future.

## Figures and Tables

**Table 1:** Surface modification of polymeric liquid separation membranes using various biomolecules.

Technique	Materials	Membrane Process	Type of Membrane	Application	Performances	Reference
Surface grafting	Arg grafted on PVA	RO	TFC	2000 ppm NaCl solution, 30,000 ppm of NaOCl and 1000 ppm of BSA	- Water flux and salt rejection increased from 52.8 L/m^2^h to 57.2 L/m^2^h and from 95.58% to 99.50%, respectively. - Chlorine resistance improved. - Antifouling property improved.	[138]
Surface grafting	3-(4-(2-((4-amino phenyl)amino)ethyl)morpholino-4-ium) propane-1-sulfonate (PPD-MEPS)	RO	TFC	NaCl solution and 300 ppm of BSA	- Water permeability enhanced from 2.40 L/m^2^h bar^−1^ to 3.81 L/m^2^h bar^−1^. - Antifouling property improved.	[139]
Chemical coupling	ε-poly-L-lysine (PL)	RO	TFC	2000 ppm of NaCl solution, 300 ppm of BSA solution, 50 ppm of DTAB solution, and 3000 ppm of HClO	- The water flux was slightly increased from 43.0 L/m^2^h to 46.3 L/m^2^h. - Antifouling property improved. - Chlorine resistance improved.	[141]
Interfacial polymerization	ABM with proteoliposomes	RO	TFC	Water reclamation process and 10 mM NaCl solution	- Water permeability increased. - Membrane stability was enhanced. - Water flux recovery rate after cleaning increased.	[142]
Covalent bonding	α-amylase and lysozyme enzyme	PDA/PEI	PES	10^8^ cfu/mL of bacterial solution	- Surface hydrophilicity improved. - *S. aureus* and *S. epidermidis* biofilms reduced by 87.5% and 94.1%, respectively.	[143]
NIPS	0.01 wt %, 0.05 wt %, and 0.1 wt % pure ginger	UF	PVDF	*S. aureus* (Gram positive) and *E. coli* (Gram negative)	- Antibiofouling property improved. - Water permeability improved from 5.07 L/m^2^h bar^−1^ to 8.82 L/m^2^h bar^−1^.	[144]
Surface Coating	Catechol/chitosan	MF	PVDF	Oil-in-water emulsions	- Water flux increased from 254 L/m^2^ h to 428 L/m^2^ h. - Antifouling property improved.	[145]
Grafting and Schiff base reaction	2-N-propyl sulfonated chitosan (PCS)	RO	PVDF	BSA solution	- The water flux increased from 86 L/m^2^h to 136.3 L/m^2^h. - The salt rejection increased from 48% to 93%. - The BSA adsorption rate decreased from 121.3 μg/cm^2^ to 29.3 μg/cm^2^. - Antifouling property was enhanced.	[146]
Evaporation casting as selective layer	CNTs/CS and CNTs-COOH/CS	Nano- composite	PSf	10 ppm of mixture of heavy metal ions	- Surface hydrophilicity improved. - Water flux increased from 0.998 L/m^2^h to 3.92 L/m^2^h. - Significantly improved the metal ion rejection rate by almost 99%.	[147]
Interfacial polymerization	0.21 g of CNC	UF	TFC	1500 ppm of NaCl and 2500 ppm of CaCl_2_	- Average water flux was enhanced from 5.9 L/m^2^h to 10 L/m^2^h and from 5.0 L/m^2^h to 6.7 L/m^2^h for NaCl and CaCl_2_ solutions, respectively. - Able to retain high salt rejection rates. - Eco-friendly nature.	[149]
Surface grafting	Dialdehyde carboxymethyl cellulose (DACMC)	RO	TFC	NaCl solution and one surfactant (200 ppm SDS and 10 ppm CTAB)	- Salt rejection slightly increased from 97.8% to 99.2%. - Antifouling property improved.	[150]
Blending and polymerization	4% of PDA	RO	PVDF	50 ppm of humic acid	- Surface hydrophilicity improved. - Antifouling property improved. - Anti-UV degradation improved.	[151]
Blending	1 wt % PDA and 0.5 wt % H_2_O_2_	UF	PES	50 ppm of humic acid	- Surface porosity improved and led to enhanced water flux from 47.06 L/m^2^h to 69.75 L/m^2^h. - Flux recovery ratio (FRR) increased from 66.94% to 94.58%. - Antifouling property improved.	[152]
Blending and polymerization	2 g of dopamine and Tris-HCl solution for polymerization reaction	UF	PVDF	50 ppm of humic acid	- Water flux increased from 17 L/m^2^h to 111 L/m^2^h. - Humic acid rejection increased from 9% to 51%. - Antifouling property improved, increasing in FRR from 54% to 92%.	[153]

## Data Availability

Not relevant.
